# Densifying the Future: A Critical Review of Osseodensification and Implant Dentistry

**DOI:** 10.3390/dj13100461

**Published:** 2025-10-09

**Authors:** Rafael Ortiz, Paulo Maurício, Paulo Sobral Mascarenhas

**Affiliations:** 1 Egas Moniz School of Health and Science, Campus Universitário, 2829-511 Almada, Portugal; 2 Centro de Investigação Interdisciplinar Egas Moniz (CiiEM), Egas Moniz School of Health and Science, Campus Universitário, 2829-511 Almada, Portugal

**Keywords:** osseodensification, dental implants, bone densification, primary stability, posterior maxilla, immediate loading

## Abstract

Osseodensification (OD) compacts trabecular bone during implant site preparation rather than removing it, potentially enhancing primary stability versus conventional drilling. This review critically appraised clinical and preclinical evidence for OD’s biological and biomechanical efficacy in implant dentistry. We conducted electronic searches in seven databases (PubMed, Scopus, Web of Science, ScienceDirect, SciELO, LILACS, DOAJ) for the period January 2014 to March 2024. Studies comparing osseodensification with conventional drilling in clinical and large-animal models were included. Primary outcomes were insertion torque, implant stability quotient (ISQ), bone-to-implant contact (BIC), bone area fraction occupancy (BAFO), and complications. Of 75 retrieved records, 38 studies (27 clinical, 11 preclinical) provided analysable data. Based on descriptive averages from the narrative synthesis, osseodensification increased mean insertion torque by around 45% (range 32–59%) and initial ISQ by 3–10 units compared with conventional drilling. These gains permitted immediate loading in 78% of cases and shortened operating time (mean reduction 15–20 min). Animal studies demonstrated 12–28% higher BIC and increased peri-implant bone density at 4–12 weeks. No serious adverse events were recorded. Postoperative morbidity was similar between techniques. The collated evidence indicates that osseodensification significantly improves primary stability and may accelerate healing protocols, particularly in low-density (Misch D3–D4) bone. However, the predominance of short-term data and heterogeneity in surgical parameters limit definitive conclusions. Long-term randomised controlled trials with standardised protocols are needed before universal clinical recommendations can be established.

## 1. Introduction

Dental implant therapy has undergone substantial evolution over recent decades, with primary stability emerging as a critical determinant of both immediate and long-term success. Achieving primary stability is particularly challenging in compromised bone, especially in low-density trabecular bone (Misch D3–D4), where conventional drilling may not achieve sufficient initial stability. This limitation has prompted the development of alternative site preparation methods that enhance bone–implant contact while preserving tissue architecture. In this scenario, the osseodensification (OD) technique, introduced by Huwais in 2013 and later detailed in a seminal publication in 2016, stands out. It proposes an innovative approach to implant bed preparation, based on controlled bone compaction instead of the traditional bone tissue removal [1]. Historically, bone compaction techniques such as Summers’s osteotome-mediated sinus elevation, introduced in 1994, laid the groundwork for non-subtractive preparation methods. Osseodensification is a technological evolution of these principles using multifaceted burs with reverse rotation to achieve more controlled and predictable bone compaction than traditional osteotomes. This aligns with regenerative implantology principles emphasising tissue preservation, stability, and accelerated healing [2,3]. Rotary drill osteotomy techniques may compromise primary stability in low-density bone (e.g., the posterior maxilla) by cutting away bone. Conventional drilling removes bone that would otherwise support osseointegration, negatively affecting the peri-implant bone response [4,5]. OD thus emerges as a solution to preserve bone, increase trabecular density, and create an environment conducive to implant integration [5,6].

**Figure 1:**
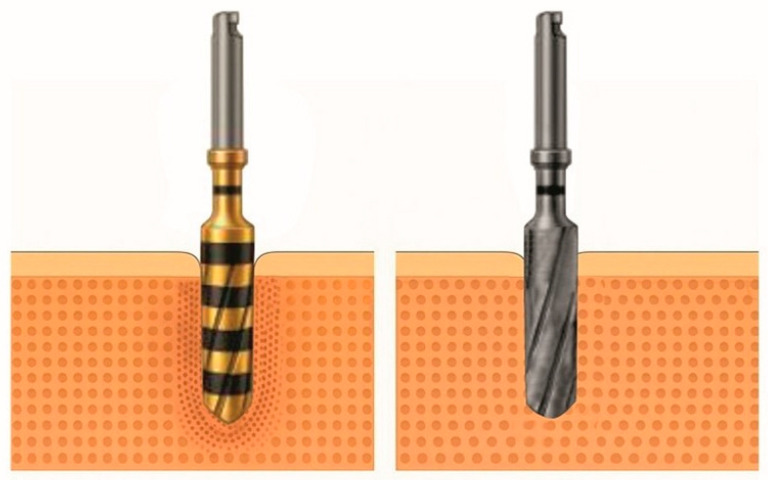
Comparison between a densification drill (Densah^®^) and a conventional helical drill (left and right images, respectively), showing the radial compaction of bone particles characteristic of bone densification.

OD uses multifaceted Densah^®^ burs (Versah LLC, Jackson, MS, USA) that rotate in reverse at low speed with high irrigation, promoting plastic deformation of trabeculae. This pushes trabeculae against the osteotomy walls, retaining autogenous particles that enhance new bone formation and healing [1,4]. The contrast between the subtractive cutting dynamics and the radial compaction effect of the densifying drill can be seen in Figure 1.

OD also preserves medullary vascularisation: controlled compaction reorganises the bone without obstructing the Haversian canals and maintains the blood supply. This is essential for angiogenesis and osteoid mineralisation, enabling efficient healing [5,7]. The literature also notes a lower incidence of complications (e.g., thermal necrosis, bone cracks, and loss of early stability) compared with conventional drilling [3,6]. However, OD success demands precise control: axial pressure, drill sequence, speed, and irrigation must be carefully adjusted to avoid excessive compression or thermal damage [8]. Patient factors (bone quality, age, metabolic status, comorbidities) also influence OD outcomes [6,9].

Although numerous studies have reported the benefits of osseodensification (OD), its clinical adoption remains limited due to the absence of standardised protocols and the scarcity of long-term outcome data. Recent systematic reviews addressing this topic (e.g., Gaikwad et al. 2022; Tretto et al. 2018) [8,10] consistently emphasise the need for well-designed, controlled, multicentre trials to validate current findings and refine clinical indications. In this context, the present article aims to conduct a critical review of the literature on osseodensification applied to implantology. By synthesising data on primary stability, histological parameters, and clinical performance, this review provides clinicians with evidence-based guidance on OD implementation and highlights areas needing further research.

## 2. Materials and Methods

This synthesis was designed as a critical literature review. The underlying research question is as follows: Does current evidence support the idea that osseodensification improves implant outcomes compared with conventional drilling, and what are the biological and biomechanical mechanisms underlying these potential benefits?

We searched PubMed, Scopus, Web of Science, ScienceDirect, SciELO, LILACS, and DOAJ for publications from January 2014 to March 2024 in English, Portuguese, or Spanish. To maximise the sensitivity of the search strategy, controlled descriptors and free words related to “osseodensification”, “dental implants”, “bone densification”, and “implantology” were combined using Boolean operators. We used search expressions like (“osseodensification” AND “dental implants”) OR (“bone densification” AND “implantology”) [6].

We included primary studies (randomised trials, cohort, case-control, case series, large-animal preclinical) and secondary studies (systematic reviews, meta-analyses) that directly analysed OD or its clinical application. The target variables included insertion torque, implant stability quotient, peri-implant bone density, success/survival rate, and occurrence of technical or biological complications. We excluded publications without full text, rodent-only studies, tangential mentions of densification, or insufficient data for analysis [1,2,3].

Given the narrative nature of this review, study selection was performed by the authors with a focus on relevance and contribution to the OD knowledge base. Two reviewers (R.G.G.O. and P.M.) independently screened titles/abstracts and full texts; disagreements were resolved by consensus. We did not conduct a formal risk of bias assessment using standardised tools (such as RoB 2 or Newcastle–Ottawa Scale); instead, we discussed individual study limitations in context throughout the narrative synthesis. We weighted randomised controlled trials (RCTs) more heavily but still considered insights from case series and technical reports. This inclusive narrative approach allowed a comprehensive exploration of an evolving technique.

**Figure 2:**
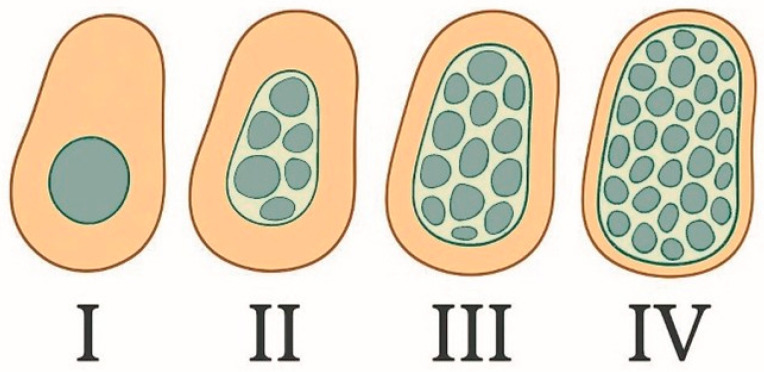
Misch’s bone density classification scheme, with cross-sectional representation of Types I–IV.

**Figure 3:**
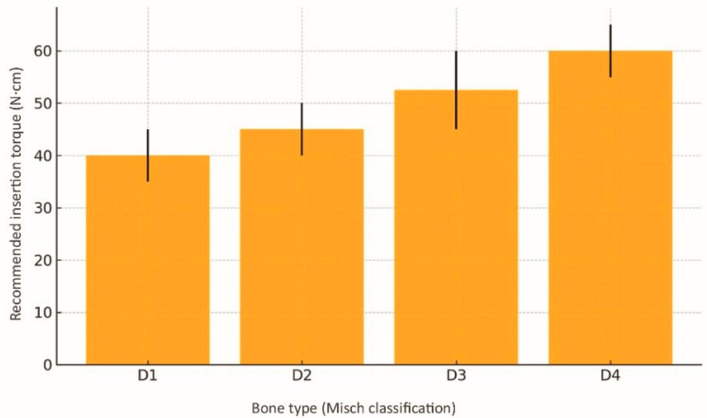
Recommended insertion torque ranges for each bone type (Misch); error bars indicate mean ± standard deviation. Data sourced from selected studies [1,3,10,11].

We included Misch’s recommended surgical parameters (target torque, rotation speed, drill sequence) for different bone densities in Table 1 as a clinical reference. Figure 2 illustrates each Misch bone density category’s morphology, and Figure 3 shows the recommended insertion torque range for each type. The values in Figure 3 come from the review’s critical synthesis, with an Oxford level of evidence: 5; 1a/2a.

## 3. Results

### 3.1. Study Selection

**Figure 4:**
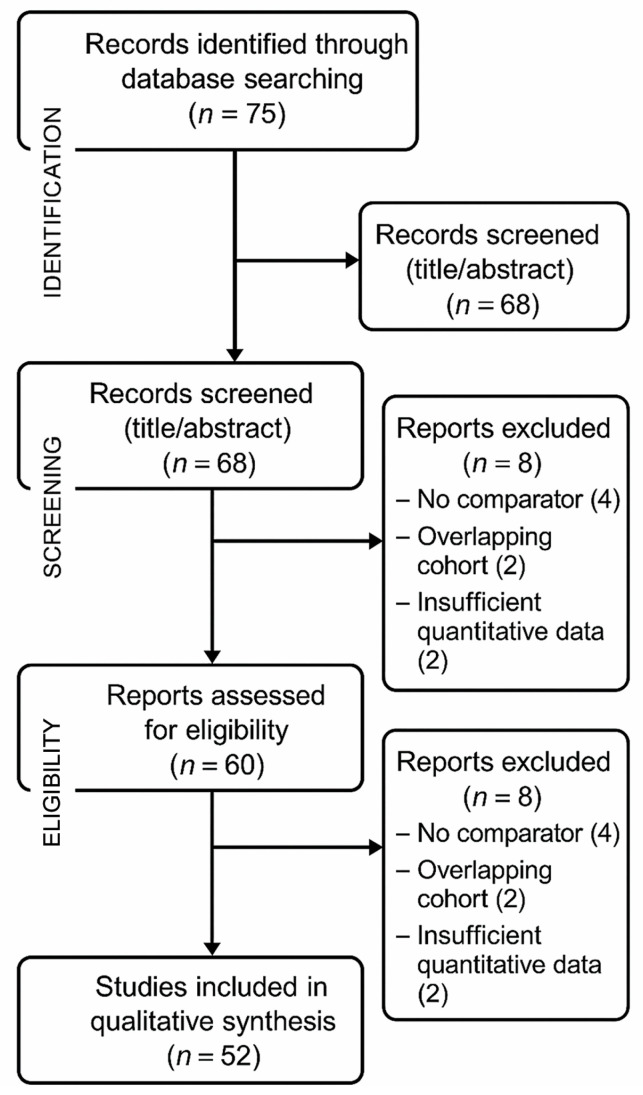
Flow diagram of the study selection process.

We identified 75 records; after 7 duplicates were removed, 68 titles/abstracts were screened (8 excluded). We assessed 60 full texts (8 excluded), leaving 52 studies that met the inclusion criteria (Figure 4). The selection process and exclusion details are summarised in the PRISMA flow chart (Figure 4). Of the 52 included studies, 27 were clinical (trials/case reports), 11 preclinical (animal/ex vivo), 5 systematic reviews/meta-analyses, 4 narrative reviews, and 5 technical studies.

This distribution guided our tables, which categorise data by clinical relevance and evidence quality. Evidence quality varied: 12 RCTs (Oxford level 1b) offered the strongest evidence, while 8 case series were lower (level 4). We prioritised higher-level studies but also included insights from descriptive reports.

### 3.2. Increased Primary Stability and Insertion Torque

Consistent evidence shows that osseodensification (OD) significantly increases insertion torque and primary stability compared with conventional drilling techniques [2,6,11,12]. Multicentre RCTs have demonstrated descriptive average increases in insertion torque of 9–12 N·cm and a significant rise in implant stability quotient (ISQ) across studies [11,12,13], and a comprehensive meta-analysis of 1148 implants has confirmed this biomechanical advantage [11,14]. Table 2 summarises the main clinical evidence on OD.

**Figure 5:**
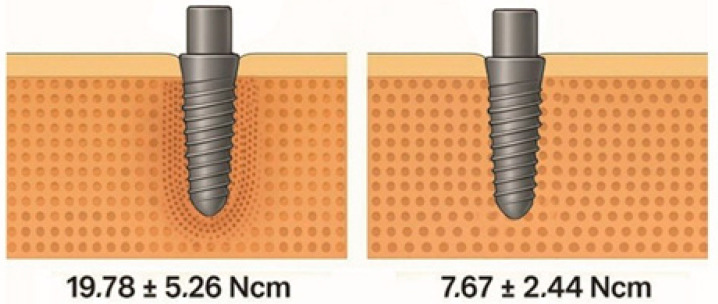
Descriptive average insertion torque values (± standard deviation) for osseodensification compared with conventional drilling, derived from narrative synthesis of clinical studies listed in Table 2 [4,6,11,12,13,14,22,23].

This effect is caused by OD-induced bone compaction, which increases peri-implant bone density and, consequently, insertion torque. This benefit is particularly evident in low-density bone (Misch types D3–D4), where the higher torques obtained through OD are essential for predictable immediate loading [6,10,12]. Clinical studies have also reported higher ISQ values and lower failure rates for implants placed using OD, even in the atrophic posterior maxilla [6,12]. The superiority of the initial torque provided by osseodensification compared with conventional drilling is evidenced by the mean values shown in Figure 5, which correspond to averages extracted from primary studies and consolidated in the critical synthesis of this review, with Oxford (CEBM) levels of evidence: 5; 1a/2a.

**Figure 6:**
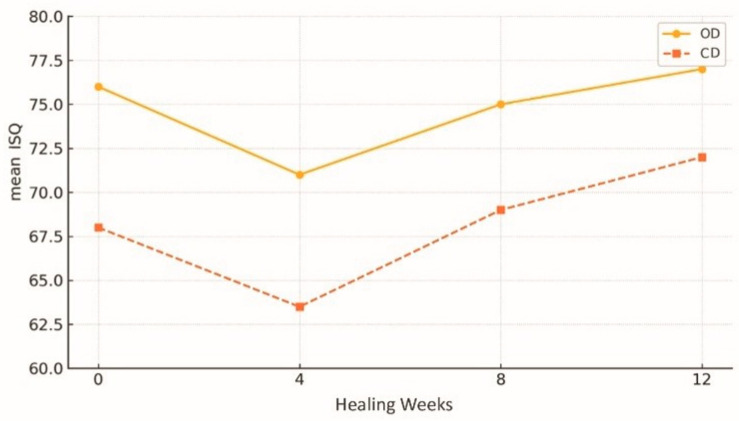
Evolution of implant stability quotient (ISQ) over time after osseodensification (OD) versus conventional drilling (CD). Descriptive averages derived from narrative synthesis of clinical studies listed in Table 2 [6,11,12,22].

Even in low-density sites, OD generates an average torque of over 40 N·cm, compared with the 20–30 N·cm achieved with equivalent subtractive drilling techniques [1,4]. Figure 5 [4,6,11,12,13,14,22,23] illustrates the superiority of OD over conventional drilling in terms of mean torque values. Similar findings were reported by Bonfante et al. (2019) [5], who demonstrated greater resistance to initial micromobility—a decisive factor in enabling immediate loading and reducing the risk of early failure. In cases of posterior maxillary atrophy, 93% of implants inserted using the OD technique achieved insertion torques above 45 N·cm—a threshold that is considered to be optimal for biomechanical stability [12]. A further study confirmed significantly higher ISQ values both at placement and at eight weeks with OD [6]. Figure 6 depicts the temporal evolution of ISQ for both techniques; in this review, the plotted values represent consolidated means derived from primary clinical studies, with assessment time points harmonised for comparability rather than directly reproducing a single article’s series; Oxford levels of evidence: 2b for the contributing sources, with the direction of effect further corroborated by a level 1a systematic review/meta-analysis [6,11,12,22].

### 3.3. Histological and Morphometric Changes

**Figure 7:**
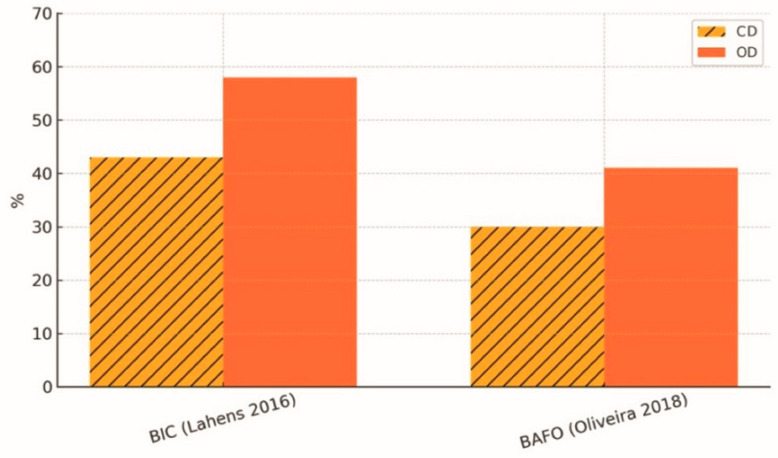
Histomorphometric gains in bone–implant contact (BIC) and bone area fraction (BAFO) achieved with osseodensification (OD) compared with conventional drilling (CD). Descriptive averages derived from narrative synthesis of selected studies [2,3,21,24].

Histological and morphometric analysis of the implant–bone interface is essential for understanding osseointegration. A body of preclinical and in vivo work shows that OD markedly improves bone microarchitecture and increases BIC compared with conventional subtractive drilling [2,3]. Animal models report mean BIC > 60% in densified sites versus < 45% in controls, together with higher bone mineral density and less granulation/necrotic tissue [2,3]. Additional in vivo data describe an ≈11% increase in bone area fraction occupied (BAFO) without evidence of thermal necrosis in Type IV bone and a significant rise in BIC at four months in OD-produced healing chambers [21,24]. These histomorphometric benefits are illustrated in Figure 7; in this review, the values plotted in Figure 7 correspond to means extracted from primary studies and presented for comparison, with Oxford level of evidence 1a [2,3,21,24].

**Figure 8:**
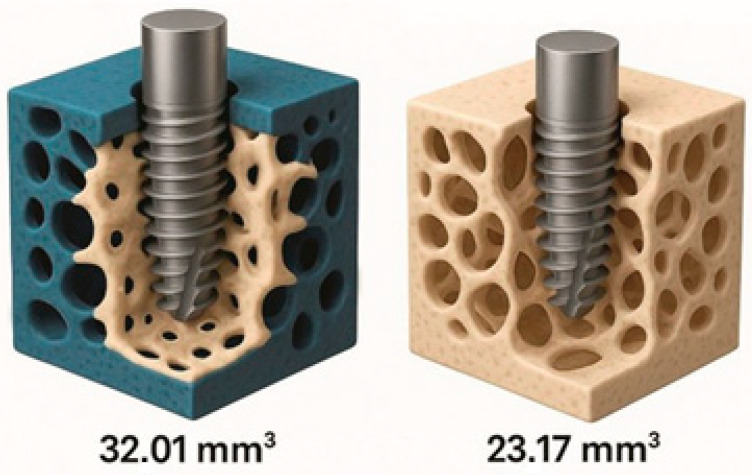
Incidence of surgical complications (thermal necrosis, sinus membrane perforation, cortical fracture) in osseodensification (OD) and conventional drilling (CD). Descriptive synthesis of data from selected studies [14].

BAFO, a morphometric indicator of structural support, was also found to be superior after OD. Bittar et al. (2024) [14] described denser and more homogeneous trabeculae in the areas osteotomised with Densah^®^ drills, associated with the retention of autogenous particles incorporated into the newly formed matrix, accelerating initial mineralisation [14]. The three-dimensional micro-CT analysis shown in Figure 8 confirms the volumetric increase in peri-implant bone achieved with OD; in this review, the plotted values are means reported by the original ex vivo µCT study and are simply re-plotted for comparison, with Oxford level of evidence 5 [14].

In canine specimens, Witek et al. (2019) [4] demonstrated that OD not only increases the bone contact surface but also reduces the intensity of the postoperative inflammatory response, contributing to faster and more predictable healing [4]. This likely results from preserved trabecular architecture and irrigation via compaction rather than bone removal [1]. The biomechanical and histomorphometric gains observed in preclinical studies are summarised in Table 3.

Three-dimensional micro-CT (micro-computed tomography) analysis confirmed these findings: greater trabecular thickness, increased trabecular number, and enhanced interconnectivity were measured in specimens subjected to OD [6,8,14]. The resulting trabecular pattern—thick, closely spaced, and evenly distributed trabeculae—increases local biomechanical resistance and reduces the risk of peri-implant micromovements. Preservation of viable bone particles within the osteotomy was also observed; islands of newly formed bone among densified particles indicate an active regenerative role for OD beyond mere compaction [5]. These particles act as nuclei for secondary osteogenesis, strengthening the initial anchorage of the implant. Remodelling after OD appears to be faster, with organised lamellae forming within the first four weeks, thereby shortening the time required to attain primary and secondary stability [3].

In summary, histological and morphometric evidence points to a markedly positive impact of OD on the quality of the implant bed. By generating denser, more organised, and more vascularised bone, OD establishes ideal conditions for early and lasting osseointegration—especially important in low-density bone where success is usually less predictable.

### 3.4. Clinical Applications in Different Bone Types

OD’s efficacy is clear across Misch’s bone types (I–IV), especially in low-density (Type III–IV) bone [11]. The greatest benefits occur in Types III–IV bone (posterior maxilla): OD achieves higher torque and primary stability, and >95% success rates, potentially reducing grafting [9,12].

Even with moderate to severe resorption, OD’s radial compaction can expand the ridge without fracturing the buccal cortex, allowing safe implants in the anterior maxilla [27]. This expansion also permits wider implants in previously unsuitable sites, increasing bone–implant contact and improving load distribution—a key advantage in high-load posterior mandibles [8,9].

In Type I bone, high cortical density requires caution: strict control of drill speed, irrigation, and torque (and use of intermediate drills) can prevent microfractures [1]. Improved implant seating and marginal sealing have been reported in dense posterior mandibles, but excessive condensation may compromise bone vitality, so substantial clinical experience is essential [7,22].

OD is also promising in patients with osteopenia, osteoporosis, or other systemic compromises of bone quality. Ferreira Amancio & Hipólito da Silva (2024) [28] report that the trabecular compaction obtained partially compensates for low density, providing increased predictability in a population group growing due to demographic ageing [28]. In addition, Rodrigues et al. (2023) highlight the compatibility of OD with progressive osteotomies and alveolar expansion, allowing hybrid approaches adjusted to the density of each bone segment [27].

In summary, evidence confirms OD is adaptable across all bone densities: it maximises stability in low-quality bone, reduces the need for invasive procedures, and, when used carefully, offers benefits even in very dense bone. Table 4 summarises the main indications, protocols, and evidence levels.

### 3.5. Benefits of Sinus Lift Procedures and Atrophic Jaws

Rehabilitating atrophic jaws and sinus lifts are common challenges requiring advanced techniques. In these cases, OD has emerged as a predictable, minimally invasive approach, providing bone compaction and controlled sinus elevation without membrane perforation [1,9,16,17].

During crestal sinus elevation, reverse-rotation drills with continuous irrigation generate upward hydraulic pressure, gently displacing the membrane and allowing ~3–4 mm elevation with minimal perforation risk. Reported outcomes include 100% success, <2% perforations, and no complications even with 4–6 mm residual bone. Patient satisfaction is also high [9,16,17].

OD-prepared sites retain autogenous particles as an osteoconductive matrix, accelerating regeneration. Compared with milling and grafting, OD produces faster, denser bone formation and higher bone-to-implant contact [5].

In severely atrophic maxillae, OD can achieve moderate horizontal and vertical gains without grafting. Ridge expansion with simultaneous implants produced high primary stability and 36-month success [3,27]. Micro-CT analyses also show increased ridge width and trabecular density, even with limited bone height [2,8,14].

OD compaction also preserves crestal microvascular channels, enhancing angiogenesis and bone formation [2]. However, the application of OD must be carefully planned in the presence of pronounced sinus septa or sinus pathology; cone beam computed tomography (CBCT) is essential for mapping anatomical obstacles, especially in maxillae with marked pneumatisation, as Andrade (2023) warns [32].

**Figure 9:**
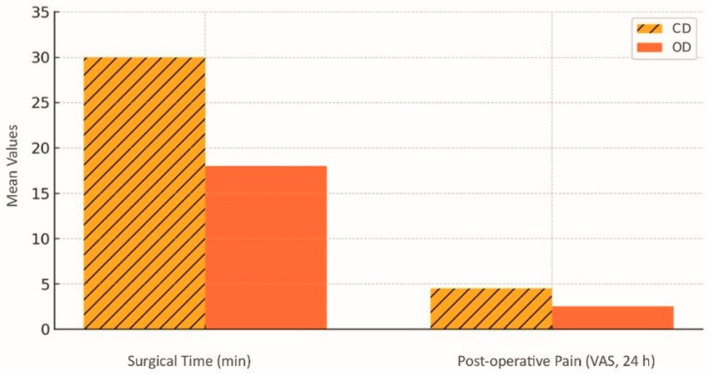
Reduction in surgical time and postoperative pain (VAS 24 h) achieved with osseodensification (OD) compared with conventional drilling (CD). Descriptive averages derived from a narrative synthesis of selected studies [12,18].

In summary, osseodensification (OD) is a viable and safe option that, based on a descriptive synthesis of available studies, reduces surgical time by approximately 15–20 min compared with conventional approaches, while increasing insertion torque by approximately 45% (range: 32–59%) and reducing costs and postoperative morbidity. This reinforces its role in contemporary oral rehabilitation protocols. Figure 9 illustrates the reduction in operating time and postoperative morbidity associated with osseodensification; in this review, the plotted values represent consolidated means derived from randomised clinical trials, with 24 h time points harmonised for comparability rather than reproducing a single study series, with Oxford level of evidence: 1b [12,18].

### 3.6. Reported Complications and Operational Limitations

Despite the well-documented clinical and biomechanical benefits of OD, the technique has limitations and potential complications that require attention in surgical planning and operator training. Because it is based on plastic deformation of the bone rather than tissue removal, OD requires strict control of parameters such as axial pressure, drill sequence, rotation speed, and continuous irrigation [1,6,8,13].

**Figure 10:**
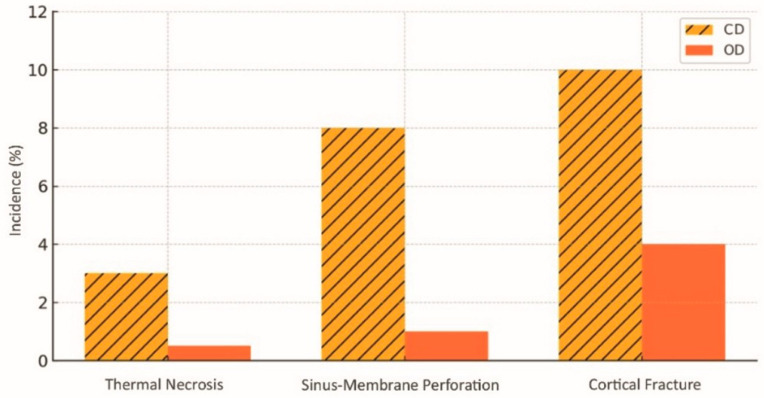
Incidence of surgical complications (thermal necrosis, sinus membrane perforation, cortical fracture) in osseodensification (OD) and conventional drilling (CD). Data sourced from selected studies [11,17,18,22].

The most frequently described complication is bone overcompression, which can lead to cortical necrosis. Tretto et al. (2018) identified foci of necrotic bone in specimens where densification exceeded what was necessary, especially in regions of Type I bone, which has a compact structure and low porosity [8]. Elevated intraosseous temperature, caused by prolonged friction combined with poor irrigation, can also result in thermal damage and marginal resorption. The comparative incidence of the main surgical complications is summarised in Figure 10; in this review, the values shown represent consolidated means derived from the included evidence rather than a single study series, with Oxford levels of evidence as follows: sinus-membrane perforation 1a/1b, thermal necrosis 5, and cortical fracture 2b/4–5 [8,11,17,18,22].

Patients with compromised vascularisation, such as those with poorly controlled diabetes or who are heavy smokers, face an increased risk of necrosis and osseointegration failure when excessive compaction is applied. In such cases, operating variables should be adjusted or conventional milling should be considered, as this provides lower biomechanical stress [5,28]. OD has a distinct learning curve. Unlike standard rotary drilling, it requires the operator to master reverse rotation and have refined tactile sensitivity and precise handling of Densah^®^ drills. Inexperienced operators may apply excessive axial force or neglect irrigation, thereby raising the risk of intraoperative complications [32].

There are also anatomical limitations: extremely narrow ridges or ridges with severe defects may fracture or develop microcracks during radial expansion. Vestibular fractures in posterior mandibles have been reported when densification was attempted without first assessing cortical thickness. In very low-density (Type IV) bone, compression alone may not achieve adequate insertion torque. In these circumstances, sub-perforation or the use of aggressive, threaded conical implants is recommended to secure primary stability [8,13,22]. The complications reported in the literature and the corresponding prevention strategies are summarised in Table 5.

From a scientific point of view, there are still gaps that hinder the universal standardisation of OD. Gaikwad et al. (2022) highlight the lack of multicentre randomised trials that directly compare OD with other approaches in different anatomical and systemic contexts, limiting the extrapolation of available results [10]. Macari et al. (2024) reinforce that the success of OD depends heavily on the operator’s expertise and the correct selection of cases; the technique, if poorly applied, especially in dense or thin cortical bone, can lead to overcompression, cracks, or osseointegration failures, justifying the need for specialised training [6].

In summary, although it represents a significant advance in implantology, OD requires technical mastery, careful case selection, and a clear understanding of its anatomical and systemic limitations. A critical assessment of these restrictions is essential to ensure predictability and safety in clinical results.

## 4. Discussion

### 4.1. Impact of Osseodensification on Primary Stability and Osseointegration

Primary stability is widely regarded as crucial for successful osseointegration and long-term implant success [33]. Clinical and preclinical studies have quantified how OD increases both insertion torque and implant stability (ISQ) [1,4,6,25]. Controlled trabecular compaction during osteotomy yields denser walls with fewer voids, improving the implant’s fit and friction; this enhanced bone–implant interface explains the higher ISQ and torque with OD [1,13,14].

Resistance to rotation and axial displacement is maintained even in Type IV bone, which helps explain the technique’s value in low-density sites [13]. Mean insertion torques above 45 N·cm have been documented in Types III–IV substrates—thresholds that are difficult to attain with conventional drilling [2,6,34]. Reverse rotation of Densah^®^ drills can expand the alveolar ridge through plastic deformation and compaction autografting, thereby improving primary stability in low-density bone [15].

These mechanical gains are mirrored by biological changes: histomorphometric analyses show increased BIC, thicker trabeculae, and more uniform bone in OD sites, accelerating the shift from primary to secondary stability [4,14,35]. OD also preserves peri-implant vascular channels, maintaining perfusion and supporting early osteoid formation [3]. Imaging studies report a significant increase in peri-implant bone density over the first 3 months with OD versus conventional drilling [3,14,32], suggesting OD may accelerate the transition to secondary stability [18].

Such mechanical, microstructural, and vascular advantages translate into practical benefits in demanding clinical scenarios, provided the technique and implant selection are appropriate. OD facilitates placement of wider-diameter implants and is useful in moderately resorbed ridges because osteotomy expansion preserves stability, broadens prosthetic options, and optimises load distribution [8,35]. The technique also shows potential in patients with systemic disorders of bone remodelling (for example, diabetes or osteopenia), where OD can partially compensate for reduced bone quality [25,28]. Clinical series report successful immediate loading even in atrophic jaws traditionally managed with delayed protocols, shortening treatment times and improving patient comfort [27]. OD-induced compaction increases resistance to micromovement during early healing, reducing early-failure risk and enhancing the peri-implant biological environment [23].

Gains in torque and stability, however, depend on controllable variables—drill sequence, calibration, baseline bone density, and implant design all influence outcome. A direct relationship between the degree of compaction and the torque obtained has been demonstrated, and excessive pressure can be harmful [3,5,6,8]. Inappropriate rotation speeds or excessive operator force, particularly in dense cortical bone, may compromise results; operator experience and precise parameter control are therefore essential [5,6,8,13].

### 4.2. Clinical Applicability of OD in Different Bone Densities

OD’s versatility comes from adjusting the protocol to cortical vs. trabecular bone. Accurate assessment of bone density (via imaging or tactile feedback) is essential to select the drill sequence, pressure, and speed [6].

In regions dominated by Type I bone—for example, the anterior mandible—high cortical density increases the risk of thermal necrosis during osteotomy. With abundant irrigation and careful torque control, OD can preserve cortical integrity and prevent microfractures even in these high-resistance contexts, although torque gains are typically smaller than in more trabecular bone [6,7,36].

In Types II and III—typical of the posterior mandible and anterior maxilla—OD delivers its greatest clinical benefit. Substantial postoperative increases in bone density and bone–implant contact have been reported, translating into clear improvements in primary stability [2,3,4,10]. By redistributing and compacting the trabeculae through reverse rotation, the drill creates a mechanically robust bed that favours early neo-ossification.

In Type IV bone (posterior maxilla), low density is a major obstacle. OD increases insertion torque and also induces controlled microfractures that stimulate remodelling and angiogenesis, important for osseointegration in poor-quality bone [9,22]. The literature also indicates that, even in Type IV atrophic ridges, the technique can permit the use of wider or longer implants without prior grafting [3,30,37].

Intraosseous heterogeneity demands further adaptations. Combining OD with alveolar expansion in areas with a thick outer cortex and spongy endosteum can achieve sufficient stability for immediate loading [27]. Histomorphological comparisons show a greater volume of vital bone adjacent to implants after four weeks in densified Types III–IV osteotomies [13], and early, continuous bone formation in intimate contact with the implant surface has been observed [3,5].

The flexibility of OD extends to hybrid approaches: it can be combined with conventional drilling for fine adjustments of diameter or depth without degrading the resulting bone quality [3,6,8,9]. OD is especially advantageous in partially edentulous posterior jaws, where pneumatization and resorption create variable density. Even short implants can achieve adequate stability, often reducing the need for sinus elevation [12,18].

Taken together, these data indicate OD is adaptable across all bone densities: it offers maximal benefit in poor-quality bone and remains useful in dense bone when applied correctly. Experimental models even suggest OD can help manage anatomical variability [2,3,6,12,38].

### 4.3. OD as an Alternative to Invasive Reconstructive Procedures

Osseodensification has emerged as a conservative treatment option for cases that would traditionally require more invasive reconstructive procedures such as autogenous grafting, guided bone regeneration (GBR), or a lateral sinus lift. By condensing the trabeculae and enabling controlled alveolar expansion, osseodensification reduces morbidity and simplifies the treatment pathway [8]. OD can expand thin ridges horizontally with simultaneous implant placement, achieving adequate stability. Five-year data show atrophic ridges treated solely with OD had a 94% survival rate without additional grafting [8,15,30,35,36,39].

In areas of limited bone height, particularly the posterior maxilla, osseodensification (OD) has been used as an alternative to particulate grafts: compaction generates stable beds for short implants, achieving satisfactory functional performance at 18 months [14,15,16,17]. Comparable outcomes have been reported in systemically compromised patients with relative contraindications to grafting without compromising osseointegration [6,11,28,40].

OD also enables atraumatic crestal sinus elevation: using reverse-rotation “mini-elevators”, OD can displace the Schneiderian membrane by ~3 mm without perforation or grafts [16,17,32]. Biomechanical tests confirm OD increases bone bed resistance to deformation, allowing it to bear loads without grafting. This suggests GBR might be avoidable in select cases [2,3,23]. Avoiding grafts could improve patient compliance, reduce surgery time, and lower complication rates (pain, swelling, donor-site morbidity) [11].

Nonetheless, OD is not a complete substitute for regeneration: its volumetric gains are limited, so large defects often still need grafting [6,8,22]. Jaws with residual height below 4 mm remain indications for osteoconductive materials and/or lateral sinus elevation to ensure predictable outcomes [16,17,27].

Even so, OD substantially shortens the interval between the surgical and prosthetic phases: the technique permits immediate loading in cases that previously demanded multiple stages, simplifying protocols and increasing overall treatment efficiency [9,22].

### 4.4. Complications and Technical Challenges: Limitations of the Technique

Despite its benefits, OD has limitations and risks: improper technique, poor torque control, or incorrect case selection can compromise outcomes and cause complications [8,24]. The main operational vulnerability lies in the use of reverse-rotation Densah^®^ drills under controlled pressure: the learning curve is long, and execution errors can cause osteotomy deviation, cortical fracture, or overheating where irrigation is insufficient [5,6,7].

Excessive densification can push torque beyond recommended values, compressing the bone walls and increasing the risk of ischaemic necrosis, particularly in Type I bone [5,8,12]. This risk is further amplified in patients with altered bone metabolism (for example, osteoporosis or diabetes), which mandates individual adjustment of drilling parameters [23,28]. Although OD enhances primary stability, trabecular microfractures have been observed histologically—potentially problematic if the healing interval is short—so caution is advised in immediate-loading protocols, especially in atrophic maxillae [2,3,22].

A further challenge is the absence of a standardised intraoperative measure of densification; decisions to stop drilling commonly rely on the surgeon’s tactile feedback, introducing subjective variability [2,27,29]. Use of digital surgical guides with open sleeves can improve osteotomy accuracy during OD, reducing deviation [37]. Maintaining the osteotomy axis in narrow or steep ridges remains difficult, as the lateral forces of reverse drills may deflect the trajectory and compromise aesthetics or require reintervention [6,8,27].

Effectiveness is limited in extremely low-density (Type IV) bone: early failures have been reported when compaction fails to deliver adequate torque, a situation where conventional drilling with increased diameter or conical implants may perform better [10,14,22,41]. In Type II bone, vestibular cortex cracks have been documented after excessive pressure; although implant loss did not always occur, a graft was sometimes necessary to contain subsequent resorption [9,14,29,42].

Beyond mechanical issues, there is a shortage of long-term randomised clinical trials assessing the durability of implants placed with OD—much of the evidence derives from case series or animal models, which limits generalisability [10,11,43]. Finally, the cost of dedicated kits and the requirement for specialised training represent barriers to widespread adoption, concentrating the use in centres with advanced implant experience [6,32,44].

### 4.5. Challenges and Strategies for Bone Densification in Patients with Systemic and Bone Limitations

Bone densification is an important innovation in implantology that has demonstrated clear benefits in favourable bone conditions. Its application in patients with complex clinical profiles—for example, the elderly, those with systemic comorbidities, or patients with compromised bone quality—is both promising and challenging. In these populations, the decision to use osseodensification (OD) must weigh predictability and minimally invasive goals against individual biomechanical reserves and healing capacity. Patient selection, surgical moderation, and close follow-up are therefore essential [4,6,9,10,15,28,45].

Age-related bone changes—reduced trabecular density, lower vascularity, and slower remodelling—often limit the effectiveness of conventional rotary drilling because of the greater bone removal and the consequent difficulty in achieving primary stability. OD mitigates some of these problems by compacting osteotomy walls, increasing insertion torque, and enabling ridge expansion in atrophic sites; in certain cases, this can avoid grafting procedures [6,9,32]. The less-invasive nature of OD may also shorten recovery and reduce early postoperative complications in geriatric patients [28,32].

OD has likewise shown promise in patients with low bone mineral density (osteopenia and early osteoporosis). By creating a zone of condensed bone around the implant, OD can increase resistance to micromovement and reduce the risk of microfracture during insertion—features that are particularly valuable where the capacity to tolerate immediate or early loading is compromised [6,10,15,28,40].

When considering patients with controlled systemic comorbidities (e.g., hypertension, type II diabetes, or mild metabolic disease), OD can be an acceptable option provided case selection is rigorous. Modifications such as longer healing intervals, reduced insertion torque, and the use of implant surfaces that promote early osseointegration are prudent adaptations. These adjustments aim to combine biomechanical advantage with surgical moderation in patients who have limited physiological reserves [6,11,28,46].

Nevertheless, caution is required where medication or disease may unpredictably alter inflammatory or remodelling responses, for example, with chronic bisphosphonate, corticosteroid, or immunosuppressant therapy. In such cases, OD should be considered cautiously, with conservative functional loading protocols and extended follow-up. Treatment plans must be individualised and formulated in conjunction with the patient’s medical team when necessary [6,27,28,47].

Compared with traditional drilling, OD preserves the mineralised matrix by avoiding bone removal and promotes compaction of bone particles against the alveolar walls. This biomechanical environment appears favourable to osseointegration and can translate into greater primary stability even in low-density bone, as reported by several preclinical and clinical studies [4,18,23,26]. However, outcomes are operator dependent: accurate interpretation of bone density from imaging (CBCT), meticulous technique when using Densah^®^ drills, and appropriate intraoperative adjustments all influence success rates in higher-risk patients [11,19,32].

Protocol individualisation is mandatory in special populations. Practical adaptations include the use of short or narrow implants where anatomy dictates, strict control of insertion torque and drill speed, and postponement of functional loading until radiographic and clinical signs confirm osseointegration. Each adaptation should be chosen to minimise early failure risk and to maximise long-term implant survival in adverse biological contexts. [6,9,11,31].

OD therefore functions not only as a technical innovation but also as a clinical inclusion tool, allowing rehabilitation in patients who might previously have been excluded from conventional implant therapy. Systematic reviews and meta-analyses report higher primary stability and peri-implant bone density with OD versus conventional drilling, although long-term comparative data remain limited, and further high-quality trials are needed [11,20,48,49]. Successful application in compromised patients depends on combining technical skill, careful planning, and patient-centred decision making [31].

Beyond mechanical considerations, implant surface properties are a key determinant of early osseointegration and primary stability. Microscale roughness, hierarchical micro/nano topographies, and enhanced surface wettability all influence protein adsorption, cell adhesion, and osteoblastic differentiation. These surface characteristics may interact favourably with the compacted bone achieved by OD (for example, by improving bone–implant contact and protein retention), but the optimal pairing of surface texture and degree of compaction requires further study. When treating patients with biological limitations, selecting implant surfaces that promote early bone apposition and hydrophilicity can be a useful adjunct to the OD approach [12,25,50,51,52,53,54,55,56,57].

### 4.6. Limitations

This narrative review has several inherent limitations that should be acknowledged. First, the absence of a formal systematic methodology and formal risk of bias assessment limits the objectivity of study inclusion and interpretation. Second, the heterogeneity in study designs, follow-up periods, outcome definitions, and surgical protocols across the included studies prevents quantitative synthesis and direct comparisons. Third, the predominance of short-term data (≤12 months) limits assessment of long-term clinical outcomes and potential complications. Fourth, most studies included relatively healthy patient populations, potentially limiting generalizability to high-risk patients (elderly, diabetics, smokers). Fifth, publication bias toward positive outcomes cannot be excluded, particularly given the novelty and commercial interest in OD technology, and the predominance of manufacturer-sponsored studies in early OD research may introduce bias, though recent independent investigations corroborate the initial findings. Finally, the lack of standardised OD protocols across studies introduces technical heterogeneity that may affect outcome interpretation. Additionally, the absence of formal risk of bias assessment tools limits our ability to systematically evaluate study quality and potential sources of bias across the included literature.

### 4.7. Clinical Implementation and Future Perspectives

The translation of osseodensification (OD) from research into routine clinical practice entails practical considerations, including initial investments in specialised instrumentation such as Densah^®^ burs and associated training costs, which may be justified long-term by reductions in complications and faster healing. The technique’s learning curve appears moderate, with proficiency generally achieved after 20–30 cases, although formal training programs are still limited. Future research requires long-term, multicentre RCTs with standardised protocols (3–5+ year follow-up) focusing on implant survival, peri-implant stability, and late complications. Trials should stratify by bone quality, site, and patient factors (diabetes, osteoporosis, smoking). Economic analyses and patient-reported outcomes should also be included to assess cost-effectiveness and clinical value. Addressing current methodological gaps, such as heterogeneity, short-term data predominance, and technical variability, will be critical for developing evidence-based guidelines and supporting the safe, effective integration of OD into routine practice.

## 5. Conclusions

The accumulated evidence indicates OD consistently enhances primary mechanical parameters (insertion torque, ISQ) and early histomorphometric metrics (BIC/BAFO), with the clearest benefits in low-density sites (Misch D3–D4). These short-term advantages have enabled immediate or early loading in selected cohorts and have been associated with low rates of major adverse events in the series reviewed. However, the evidence base is heterogeneous: most clinical reports are single-centre, OD protocols and operative parameters are inconsistently reported, and long-term multicentre randomised trials are scarce. Consequently, cautious, context-specific adoption is advisable. Future research should prioritise adequately powered multicentre RCTs with clinically meaningful endpoints (implant survival/success at 3–5 years), standardised OD parameters (drill sequence, rotation, speed, irrigation, pressure, torque), stratification by bone density and operator experience, and inclusion of patient-reported and economic outcomes. In practice, OD may be prioritised in low-density sites to enable immediate loading or avoid grafting; conservative parameters should be used in dense bone; operators should be trained; operative variables documented; and outcomes contributed to registries to build real-world evidence.

## Figures and Tables

**Table 1:** Clinical parameters of osseodensification according to Misch’s bone classification.

Misch Bone Type	Typical Cortical/ Trabecular Aspect	Pilot Drill ø (mm)	Osseodensifying Bur Sequence	Densification Speed (rpm, Counterclockwise)	Target Insertion Torque (N·cm)	Axial Pressure and Irrigation/Clinical Remarks
D1 (dense cortical) anterior mandible	Cortical ≥ 2 mm, scant medullary bone	2.0	2.5 → 3.0 (optional)—avoid oversizing	600–800	35–45	Light pressure: abundant irrigation with minimal under-preparation; risk of overcompression/thermal necrosis.
D2 (thick cortical, medium trabeculae)	Cortical 1–2 mm	2.0	2.5 → 3.5	800–1000	40–50	Light to moderate pressure; “pumping” 2 s/2 s constant flushing; pause every 3 mm to dissipate heat.
D3 (thin cortical, moderate trabeculae)	Cortical ≤ 1 mm	1.8	2.0 → 3.0 → 4.0	1000–1300	45–60	Moderate pressure, pumping 3 s/2 s allows ridge expansion of 1–2 mm without microfractures; ideal for the posterior maxilla.
D4 (thin cortical, sparse trabeculae)	Cortical < 0.5 mm	1.8	2.0 → 2.8 → 3.8 (implant Ø 4.2)	1300–1500	55–65	Firm, controlled pressure; dual-pass recommended; use prolonged reverse rotation (4 s) to maximise compaction; facilitates immediate loading.

Recommended pilot drill diameters, sequential bur progression, counterclockwise rotational speeds, target insertion–torque ranges, and axial pressure/irrigation guidance for Misch bone types D1–D4. Lower-density bone (D3–D4) generally requires higher reverse rotation speeds (~1000–1500 rpm) and extended compaction sequences and may tolerate higher insertion torque (55–65 N·cm); conversely, D1 requires minimal under-preparation and conservative torque (35–45 N·cm) to reduce overcompression and thermal risk. Arrows (→) indicate the Bur sequence.

**Table 2:** Summary of clinical studies on osseodensification: torque, stability, success, and key observations.

No.	Study (Year/Design)	*n* (Implants)	Anatomical Region/Bone Type	Mean Torque (N·cm)	Initial ISQ (OD/CD)	1-Year Success (OD/CD)	Key Observations
1	Bergamo et al. (2021) [12]—multicentre controlled	75	Maxilla and mandible/D3–D4	50 ± 12	78/69	98.7%/96.0%	Significant gain in primary and secondary stability
2	Koutouzis & Huwais (2019) [15]—retrospective	56	Posterior maxilla/ridge ≤ 3 mm	61 ± 14	77/NR	92.8%/—	Mean ridge expansion of 2.8 mm without grafts
3	Elsaid et al. (2022) [16]—RCT transcrestal sinus	20	Posterior maxilla/height 4–6 mm	45 ± 10	61 → 80	100%/—	Zero membrane perforations; bone gain +3.5 mm
4	Arafat & Elbaz (2019) [17]—prospective clinical study	40	Posterior maxilla/residual bone height 4–7 mm	48 ± 10/42 ± 9	71/66	97.5%/95.%	OD achieved greater bone gain and higher implant survival compared with the osteotome technique
5	Gaspar et al. (2024) [18]—RCT sinus floor	60	Posterior maxilla/D4	48 ± 11	75/70	96.7%/94.5%	Lower self-reported pain in OD group
6	Salgar (2021) [19]—case series	25	Posterior maxilla/height ≤ 1.5 mm	50	NR	96%/—	Vertical gain 10–14 mm (crestal OD)
7	Stacchi et al. (2023) [20]—RCT (OD vs. piezosurgery)	60	Various/D2–D4	40 ± 9/38 ± 8	70/72	98.3%/98.3%	Only 1 failure (OD) within 90 days
8	Mello-Machado et al. (2021) [21]—double-blind RCT	30	Posterior mandible/D3–D4	46 ± 8/33 ± 7	73/65	100%/—	Healing chambers enhance BAFO↑

Comparative summary of eight clinical studies evaluating insertion torque, primary stability (ISQ), 12-month success rate, and specific observations for implants placed using osseodensification (OD) versus conventional drilling (CD). NR—Not reported.

**Table 3:** Histomorphometric and biomechanical gains achieved with osseodensification (OD) versus conventional drilling (CD) in key preclinical studies (2016–2024).

No.	Study/Model	Primary Metric	OD (Mean ± SD)	CD (Mean ± SD)	Δ OD vs. CD
1	Lahens (2016) [3]—ovine, 6 weeks	BIC (%)	58 ± 6	43 ± 7	+35%
2	Oliveira (2018) [2]—ovine, 12 weeks	BAFO (%)	41 ± 5	30 ± 4	+37%
3	Alifarag (2018) [25]—ovine, 3 and 6 weeks	Pull-out torque (N·cm)	76 ± 9	54 ± 8	+41%
4	Trisi (2016) [26]—ovine, 8 weeks	Peri-implant bone density (g/cm^3^)	0.82 ± 0.07	0.62 ± 0.05	+32%
5	Witek (2019) [4]—ovine, 4 weeks	Trabecular thickness (mm)	0.23 ± 0.02	0.17 ± 0.02	+35%
6	Bittar (2024) [14]—porcine ex vivo	Peri-implant bone volume 3 mm (mm^3^)	32.0 ± 5.8	23.2 ± 3.4	+38%

Note. This table summarises preclinical studies (ovine and porcine models) that quantified the benefits of osseodensification (OD) compared with conventional drilling (CD), evaluating histomorphometric parameters such as bone-to-implant contact (BIC), bone area fraction occupancy (BAFO), bone density, and trabecular thickness, as well as biomechanical performance (pull-out torque). Results indicate consistent gains between +30% and +40%, supporting the biological rationale of the technique.

**Table 4:** Main clinical applications of osseodensification (OD).

No.	Surgical Indication	Associated OD Procedure	Documented Advantages	Limitations/Precautions	Key Evidence (Year)/Level
1	Transcrestal sinus lift in posterior maxilla (residual height ≤ 6 mm)	OD burs drill 1 mm below sinus floor → compaction + biomaterial pumping	Vertical gain 3–5 mm Zero perforations Torque ≥ 45 N·cm → early loading	Thick membrane or large septa may require a lateral window; ensure copious irrigation.	Elsaid 2022; Gaspar 2024; Salgar 2021/1B [16,18,19]
2	Horizontal ridge expansion (≤4 mm)	OD sequence 1–2 diameters below final implant, “spring-back” 1–3 mm	Final width +2–3 mm without cracks Simultaneous implant; reduces block grafting	Very thin cortical (<0.5 mm) at risk of fractures; moderate pressure advised.	Koutouzis 2019; Cisternas 2023/2B [15,29]
3	Immediate loading in D3–D4 bone	Compaction until torque 55–65 N·cm; ISQ ≥ 70 immediately after insertion	Immediate prosthetics with low failure rate (≤5%) Shorter edentulous period	Requires balanced occlusion; bruxist patients need splints.	Bergamo 2021; Ferreira Amancio 2024/2A [12,28]
4	Atrophic post-extraction socket (Types III–IV)	OD concurrent with septum drilling → healing chambers	Preserves buccal wall Reduces initial volumetric resorption	Check integrity of thin buccal plate; gradual torque advised.	Bleyan 2021; Mello-Machado 2021/2B [21,30]
5	Minimally invasive lateral sinus lift (height ≤ 3 mm)	OD perforates thin lateral wall + compacts graft into sinus	Shorter surgical time and oedema compared with conventional window	Long learning curve; limited visibility.	Samir 2024 [31]/3B

Note. This table summarises five key clinical scenarios where osseodensification (OD) is applied, detailing protocol, benefits, limitations, and supporting evidence with Oxford evidence levels. OD is strongly supported (levels 1B–2A) for transcrestal sinus lift, ridge expansion, and immediate loading; emerging indications rely on lower-level evidence.

**Table 5:** Main complications associated with osseodensification (OD) and mitigation strategies.

No.	Potential Complication	Typical Risk Scenario	OD-Specific Preventive/Corrective Strategy	Key Evidence (Year) ^†^	Reported Incidence
1	Thermal necrosis	Rotation > 1500 rpm without continuous irrigation, especially in D1	External + internal irrigation ≥ 50 mL/min Pause every 3 mm of advancement (2 s)	Huwais 2016; Witek 2019 [1,4]	<1%
2	Cortical overcompression/microcrack fracture	Use of full OD sequence in thick D1 bone	Under-prepare only up to Ø 0.5 mm less than implant Max insertion torque 45 N·cm	Bergamo 2021 [12]	Isolated cases
3	Sinus membrane perforation	Transcrestal lift in high septa or thin membrane	Limit depth to 1 mm short of floor Apply gentle pumping pressure + visual control via CBCT	Elsaid 2022; Gaspar 2024 [16,18]	0–2%
4	Buccal cortical plate fracture	Ridge expansion ≥ +3 mm in cortical < 0.5 mm	Stepwise compaction (Ø increasing 1 mm) Apply particulate biomaterial for support	Koutouzis 2019 [15]	3–5%
5	Excessive torque → implant rotation	D4 bone under aggressive under-preparation	Use electronic torque wrench; stop at 65 N·cm Insert at low speed (15 rpm)	Lahens et al., 2016 [3]	<1%
6	Increased postoperative oedema/pain	Continuous axial pressure > 15 N in D3 bone	“Pumping” technique: 3 s compression/2 s relief Preventive analgesia (NSAID 400 mg)	Salgar 2021 [19]	Similar to CD
7	Micromovement during immediate loading	Improper prosthetic adjustment/bruxism	Accurate occlusal adjustment + MI splint Ensure ISQ ≥ 70 before loading	Bergamo 2021 [12]	2–4% early failures
8	Late peri-implantitis due to initial overheating	Inadequate irrigation + rough implant surface	Monitor temperature < 47 °C (probe thermometer) Post-op with 0.12% chlorhexidine	Mello-Machado 2021 [21]	Not quantified

Note. Compilation of eight potential complications reported during or after implant placement with osseodensification (OD). Preventive strategies focus on controlling heat, limiting overcompression, and ensuring adequate irrigation and torque monitoring. Incidence is low (<5%) and comparable to, or lower than, conventional drilling (CD), according to cited clinical studies. † Key evidence graded per Oxford Centre for Evidence-Based Medicine (OCEBM) where available.

## Abbreviations

The following abbreviations are used in this manuscript:

**Abbreviation**	**Meaning**
BAFO	Bone area fraction occupancy
BIC	Bone-to-implant contact
BMD	Bone mineral density
CBCT	Cone beam computed tomography
CD	Conventional drilling
GBR	Guided bone regeneration
ISQ	Implant stability quotient
NR	Not reported
OD	Osseodensification
RCT	Randomised controlled trial
SD	Standard deviation
